# Comprehensive analysis of expressed sequence tags from cultivated and wild radish (*Raphanus* spp.)

**DOI:** 10.1186/1471-2164-14-721

**Published:** 2013-10-21

**Authors:** Di Shen, Honghe Sun, Mingyun Huang, Yi Zheng, Yang Qiu, Xixiang Li, Zhangjun Fei

**Affiliations:** 1 Institute of Vegetables and Flowers, Chinese Academy of Agricultural Sciences, Beijing 100081, China; 2 Boyce Thompson Institute for Plant Research, Cornell University, Ithaca, NY 14853, USA; 3 National Engineering Research Center for Vegetables, Beijing Academy of Agriculture and Forestry Sciences, Beijing 100097, China; 4 U.S. Department of Agriculture/Agriculture Research Service, Robert W. Holley Centre for Agriculture and Health, Ithaca, NY 14853, USA

**Keywords:** Radish, EST, SNP, SSR, Comparative analysis, Whole genome duplication, Phylogenetic relationship

## Abstract

**Background:**

Radish (*Raphanus sativus* L., 2*n* = 2× = 18) is an economically important vegetable crop worldwide. A large collection of radish expressed sequence tags (ESTs) has been generated but remains largely uncharacterized.

**Results:**

In this study, approximately 315,000 ESTs derived from 22 *Raphanus* cDNA libraries from 18 different genotypes were analyzed, for the purpose of gene and marker discovery and to evaluate large-scale genome duplication and phylogenetic relationships among *Raphanus* spp. The ESTs were assembled into 85,083 unigenes, of which 90%, 65%, 89% and 89% had homologous sequences in the GenBank nr, SwissProt, TrEMBL and Arabidopsis protein databases, respectively. A total of 66,194 (78%) could be assigned at least one gene ontology (GO) term. Comparative analysis identified 5,595 gene families unique to radish that were significantly enriched with genes related to small molecule metabolism, as well as 12,899 specific to the Brassicaceae that were enriched with genes related to seed oil body biogenesis and responses to phytohormones. The analysis further indicated that the divergence of radish and *Brassica rapa* occurred approximately 8.9-14.9 million years ago (MYA), following a whole-genome duplication event (12.8-21.4 MYA) in their common ancestor. An additional whole-genome duplication event in radish occurred at 5.1-8.4 MYA, after its divergence from *B. rapa*. A total of 13,570 simple sequence repeats (SSRs) and 28,758 high-quality single nucleotide polymorphisms (SNPs) were also identified. Using a subset of SNPs, the phylogenetic relationships of eight different accessions of *Raphanus* was inferred.

**Conclusion:**

Comprehensive analysis of radish ESTs provided new insights into radish genome evolution and the phylogenetic relationships of different radish accessions. Moreover, the radish EST sequences and the associated SSR and SNP markers described in this study represent a valuable resource for radish functional genomics studies and breeding.

## Background

Expressed sequence tags (ESTs), which are created by partially sequencing randomly isolated gene transcripts and converting them into cDNAs [1], provide a valuable information source for investigating a wide variety of genetic characteristics of a species. Since they represent the expressed portion of a genome, ESTs have been reported to play significant roles in accelerating gene discovery, improving genome annotation [2,3], uncovering whole genome duplication events and facilitating evolutionary analysis [4,5]. Furthermore, EST analysis represents an effective means to for rapidly identify transcripts involved in specific biological processes [6,7].

EST collections also represent a valuable resource to identify simple sequence repeat (SSR) and single nucleotide polymorphism (SNP) markers. In recent years, SSR markers have been increasingly used to construct high-density genetic maps and to identify quantitative trait loci (QTL) associated with economically important crop traits [8,9]. While SNPs typically provide more useful markers in many basic and applied research areas, such as population genetics, gene discovery, plant breeding and germplasm identification [10-12], the steps from sequencing and SNP discovery to SNP marker design and validation are generally lengthy and expensive. As a consequence of the rapid development of next generation sequencing technologies and progress with genome and cDNA sequencing projects, extremely large numbers of ESTs are becoming publicly available. Some of these sequence resources have been already exploited for the development of molecular markers such as SSRs [13] and SNPs [14,15], which have value in both elucidating phylogenetic relationships and facilitating breeding programs.

Radish (*Raphanus sativus* L., 2*n* = 2× = 18), an economically important root vegetable crop that is grown and consumed worldwide, belongs to Brassicaceae and is closely related to *Brassica rapa* and the experimental model plant *Arabidopsis thaliana*. Its fleshy, edible root varies in shape (round, oval or elongated), size (from one-half inch in diameter to one-half feet in length) and color (e.g. white, pink, red and purple) [16]. Although there are no detailed archeological records describing the early history of radish cultivation, it has been suggested that it was domesticated in Europe in pre-Roman times [17,18]. However, the origin of cultivated radish is still debated. *R. raphanistrum*, the wild species of *R. sativus*, includes three subspecies: subsp. *maritimus*; subsp. *Raphanistrum*; and subsp. *landra*. Most reports suggest that *R. sativus* L. originated from *R. raphanistrum* L., but it has also been proposed that *R. sativus* resulted from hybridization between *R. maritimus* and *R. landra*, or alternatively that it derived from interspecific hybridization between a variety of parental species [18-20].

Despite its agricultural importance as a root vegetable crop, radish has only recently been analyzed using genomic and functional genomic approaches. For example, transcriptome studies have been reported, with a focus on SSR marker development [13] and expression profiling in response to lead exposure [21]. There are currently more than 300,000 publicly available ESTs in the NCBI dbEST database [22], but this large-scale EST dataset has not been characterized in detail, and has primarily been used to derive SSR markers for genetic map construction. In order to integrate and utilize these data efficiently, and to gain more insights into the biology and genome evolution of radish, these radish ESTs were first clustered and assembled into 85,083 unigenes. This unigene set was then extensively annotated. Comparative genomic analysis of radish, *A thaliana* and *B. rapa* were performed in order to elucidate the functional and evolutionary processes that act on their respective genomes. Furthermore, putative SSR and SNP markers were identified from these ESTs and the phylogenetic relationships between the different radish genotypes were inferred. This information provided new insights into the biology of major agronomic traits of radish, as well as its genome evolution and the phylogenetic relationships between different genotypes.

## Results and discussion

### EST assembly and annotation

A total of 314,799 *Raphanus* ESTs were collected from the NCBI dbEST database. After removing low quality and contaminating sequences, 311,021 ESTs were obtained. Of these, 149,092 were from cultivated radish (*R. sativus*) and 161,929 from wild radish (*R. raphanistrum*), comprising subsets from the three subspecies: subsp. *raphanistrum* (80,380 ESTs); subsp. *landra* (41,398 ESTs); and subsp. *maritimus* (40,151 ESTs). These ESTs were generated from 22 different *Raphanus* cDNA libraries derived from 18 different accessions (Table 1). Different radish organs/tissues were sampled to construct these cDNA libraries, including cotyledons, hypocotyls, roots, root axes, leaves, flowers, whole buds and whole seedlings.

**Table 1:** **Description of *Raphanus *** **cDNA libraries and summary of *Raphanus *** **ESTs**

**No.**	**Library name**	**Species**	**Tissue/organ**	**No. of ESTs**
1	ATR-4H	*Raphanus sativus* cv Scarlet Globe	Root	61
2	ATR-24H	*Raphanus sativus* cv Scarlet Globe	Root	13
3	3 day old radish seedling	*Raphanus sativus* cv National	Cotyledon	19
4	*Raphanus sativus* differential display library	*Raphanus sativus* cv Nau-Yh, Nau-BYC, Nau-Dy13	Shoot apex and leaf	24
5	*Raphanus sativus* leaf cDNA library after bolting	*Raphanus sativus* cv Zaosheng60tian	Leaf	12
6	RR1(CS)	*Raphanus raphanistrum* subsp. *maritimus*	Whole seedling (with 1 set of true leaves), buds, and anthers	40,151
7	RR2(MS)	*Raphanus raphanistrum* subsp. *raphanistrum*	Whole seedling (with 1 set of true leaves), buds, and anthers	39,314
8	RR3(NY)	*Raphanus raphanistrum* subsp. *raphanistrum*	Whole seedling (with 1 set of true leaves), buds, and anthers	41,066
9	RR4(PB)	*Raphanus raphanistrum* subsp. *landra*	Whole seedling (with 1 set of true leaves), buds, and anthers	41,398
10	RS1(AR)	*Raphanus sativus* var. *oleiformis*	Whole seedling (with 1 set of true leaves), buds, and anthers	39,802
11	RS2(RS)	*Raphanus sativus* cv Early Scarlet Globe	Whole seedling (with 1 set of true leaves), buds, and anthers	40,812
12	RS3(RT)	*Raphanus sativus* cv Rat-Tail Radish #3870	Whole seedling (with 1 set of true leaves), buds, and anthers	41,027
13	*Raphanus sativus* bud	*Raphanus sativus*	Whole bud	15
14	Subtraction library radish hypocotyl cDNA	*Raphanus sativus* cv Sakurajima Daikon var. *niger*	Hypocotyl	163
15	Salt stress treated wild radish leaf library	*Raphanus sativus* var. *niger*	Leaf	257
16	Subtracted radish root cDNA library (*Raphanus sativus* Kosena)	*Raphanus sativus* cv Kosena	Root axis	140
17	Subtracted radish root cDNA library (*Raphanus sativus* Taibyousoubutori)	*Raphanus sativus* cv Taibyousoubutori	Root axis	70
18	*Raphanus sativus* GSK3-1 flower	*Raphanus sativus* cv GSK3-1	Flower	5,104
19	*Raphanus sativus* GSK3-1 leaf	*Raphanus sativus* cv GSK3-1	Leaf	6,720
20	*Raphanus sativus* GSK3-1 root	*Raphanus sativus* cv GSK3-1	Root	6,319
21	*Raphanus sativus* GSK3-1 seedling	*Raphanus sativus* cv GSK3-1	Seedling	8,454
22	Subtracted radish root (cv. Fuyudorishougoin) DNA library	*Raphanus sativus* cv Fuyudorishougoin	Root axis	80

The ESTs were assembled *de novo* into 85,083 unigenes with an average length of 822 bp, of which 33,322 were singletons with an average length of 594 bp and 51,761 were contigs with an average length of 970 bp. The distribution of the number of EST members in the radish unigenes is listed in Table 2. A total of 6,404 (~8%) of the unigenes had more than 10 EST members and they represented 41% of the total number of EST reads.

**Table 2:** Distribution of number of ESTs in radish unigenes

**No. EST members**	**No. unigenes**	**No. ESTs in unigene**
1	33,322	33,322
2	22,758	45,516
3	7,451	22,353
4	5,668	22,672
5	3,023	15,115
6	2,446	14,676
7	1,661	11,627
8	1,306	10,448
9	1,044	9,396
10	851	8,510
11-30	4710	78,071
31-50	649	24,663
51-70	140	8,220
71-90	33	2,593
91-110	8	800
>110	13	3,039

To functionally annotate the radish unigenes, their sequences were compared against the GenBank non-redundant (nr) protein database using the BLAST program. A total of 76,156 (90%) radish unigenes had hits in the nr database, indicating that a very high percentage of radish unigenes could be assigned a putative function. The radish unigenes were also compared against the UniProt/SwissProt, UniProt/TrEMBL and Arabidopsis (TAIR version 10) protein databases, which yielded 54,959 (65%), 75,427 (89%) and 76,042 (89%) matches in these three databases, respectively.

**Figure 1:**
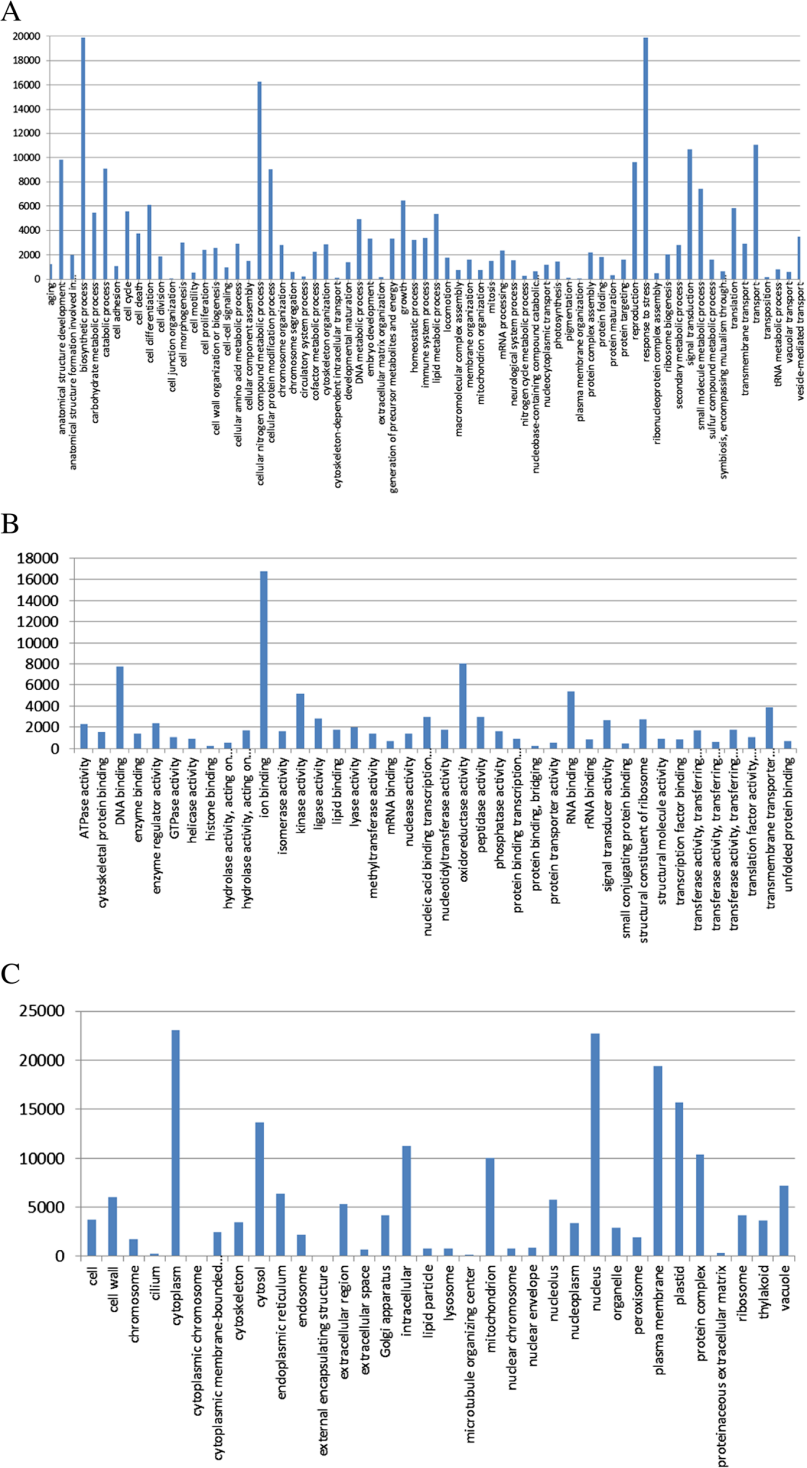
Functional classification of radish unigenes within the category of biological process (A), molecular function (B) and cellular component (C).

A Gene Ontology (GO) analysis of the radish unigenes was then undertaken. A total of 66,194 (78%) unigenes were associated with at least one GO term, of which 58,419 (69%) were assigned at least one GO term in the cellular component category, 56,634 (67%) in the biological process category and 56,389 (67%) in the molecular function category, while 47,475 (56%) were annotated with GO terms from all three categories. Go slim analysis was then performed to classify the radish unigenes into different functional categories according to the GO annotations. Biosynthetic process, ion binding and cytoplasm were the most abundant GO terms within the biological process, molecular function, and cellular component categories, respectively (Figure 1). In addition, cell differentiation, reproduction, and growth were also among the most highly represented groups within the biological process category, consistent with the fact that the majority of radish ESTs were derived from whole seedlings, buds and anthers. In addition, genes involved in other important biological processes, such as stress responses and signal transduction, were also identified.

Transcription factors (TFs) are an important and diverse class of regulatory proteins that can initiate and regulate gene transcription, and that collectively coordinate gene expression in different cell types and during development. In the present study, a thorough screen of the radish unigene dataset was performed to identify putative TFs using iTAK [23]. A total of 2,809 TFs were predicted and systematically grouped into 56 TF families (Additional file 1 and Additional file 2). The MYB family was the most abundant, comprising 277 genes, followed by the bHLH (209), bZIP (187), AP2-EREBP (178) and NAC (145) families. Identification of these TFs from radish provides a useful resource to help researchers to gain a better understanding of the intricate relationships between transcription factors and the major agricultural traits of radish.

### Comparative analysis of gene sets between radish and other plants

The 85,083 radish unigenes were compared to the complete protein sequence sets of rice, grape, Arabidopsis and *Brassica rapa* using the BLAST program. At an E-value cutoff of 1e-5, approximately 90% and 88% of radish unigenes matched proteins of *Brassica rapa* and *A. thaliana*, respectively, which belong to the same family of radish, while 79% and 77% matched proteins from grape and rice, respectively.

**Figure 2:**
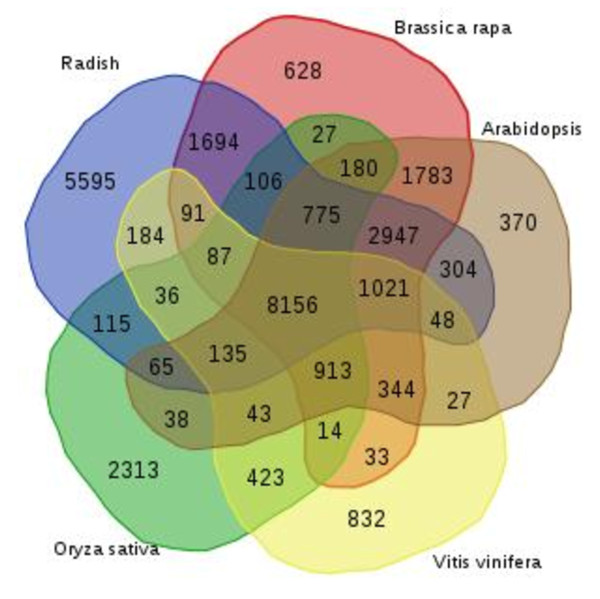
**Venn diagram of ortholog group distribution in radish, Chinese cabbage (*Brassica rapa*), Arabidopsis, grape (*Vitis vinifera*) and rice (*Oryza sativa*).** Numbers in each section indicate the numbers of ortholog groups.

Gene family clustering analysis was performed for the above five plant species. A total of 172,156 gene sequences from the five species were clustered into 29,327 gene families (Figure 2). Of these, 8,156 were shared by all five species, and so may represent highly conserved gene families among dicotyledonous and monocotyledonous plants. Of the 85,083 radish unigenes, 54,195 were divided among 21,359 of these gene families, while 5,595 gene families were unique to radish. The analysis also revealed that 12,899 gene families were unique to the Brassicaceae family (radish, *B. rapa* and *A. thaliana*). Functional analysis using GO terms showed that gene families that were specific to the Brassicaceae family were significantly enriched with GO terms involved in response to hormones, such as auxin and gibberellins, and seed oil body biogenesis (Additional file 3). These observations can be associated with well-known characteristics of the Brassicaceae since bolting, one of the most important traits of the family, can be induced by gibberellins [24,25], and seeds of the Brassicaceae contain a high oil content, which makes them good candidates for producing feedstock oils for biodiesel. The oil body is a unique oil storage organelle, consisting mainly of triacylglycerol (TAG) surrounded by a layer consisting of phospholipids and the protein oleosin [26]. It has been demonstrated that the quantity and size of oil bodies correlate well with the oil content of rapeseed and that oil body biogenesis may be coupled with the embryogenesis [27]. Gene families specific to radish were significantly enriched with GO terms related to small molecule metabolic processes, including monosaccharide and organic acid metabolism (Additional file 4). This observation aligns well with the most important agronomic product of radish, the fleshy tap root, since during taproot development the carbohydrate content, including total soluble sugars, sucrose and fructose decrease at the onset of taproot swelling, [28].

### Whole genome duplications in radish

**Figure 3:**
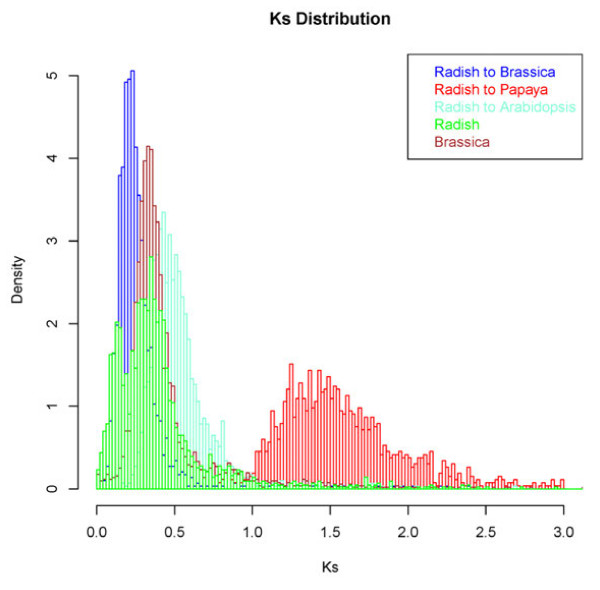
**Distribution of synonymous nucleotide substitution (Ks) rates between homologous gene pairs within radish (green), *Brassica rapa *** **(brown) and between radish and *B. rapa *** **(blue), radish and Arabidopsis (cyan), and radish and papaya (red).**

Whole genome duplication (WGD) is common in angiosperms and is regarded as the major evolutionary force that gives rise to novel gene functions and transcriptome sequences have been successfully used to identify WGD events [4,5]. In the present study, gene families were constructed using proteins from four species, *A. thaliana*, *Brassica rapa*, Carica papaya (papaya) and radish, which all belong to the order Brassicales. A total of 1,472 gene families with a single gene in each species were used to date speciation events and homologous pairs with best reciprocal matches within each species were used to identify WGD events. A total of 1,422, 2,594, 630 and 2,175 pairs were identified for *A. thaliana*, *B. rapa*, papaya and radish, respectively. Ks values for these homologous gene pairs were calculated. The peaks of the Ks values corresponding to the WGD events in *A thaliana*, *B. rapa*, and papaya (Figure 3 and Additional file 5) are consistent with those presented in the PGDD database [29], indicating the robustness of the approach.

Based on the distribution of Ks values of homologous genes, two recent WGD events in radish, α and β, were identified, which were estimated to have occurred approximately 5.1-8.4 and 12.8-21.4 million years ago (MYA), corresponding to the peaks of Ks at 0.13 and 0.35, respectively (Figure 3). The β event occurred before the divergence of radish and *B. rapa* approximately 8.9-14.9 MYA and is shared by *Raphanus* and *Brassica*, while the α event occurred after the divergence and is *Raphanus* specific*.* The analysis also indicated that the common ancestor of radish and *B. rapa* diverged from *A. thaliana* about 15.9-27.8 MYA, which is consistent with previous reports [30,31].

### Identification of simple sequence repeats (SSRs) and single nucleotide polymorphisms (SNPs)

Both SSRs and SNPs are valuable markers for genetic mapping and marker-assisted breeding. SSR and SNP markers derived from EST sequences, which directly link to expressed genes, have been widely used in linkage map construction and genetic mapping of QTLs associated with important agronomic traits [11,32]. In the present study, a thorough screen of the radish unigene dataset for the presence of SSRs was performed. A total of 13,570 SSR motifs were discovered in 12,403 radish unigenes. The major types of the identified SSRs were tri-nucleotide (6,651) and di-nucleotide (5,918), accounting for 49% and 44% of the SSRs, respectively, followed by tetra-nucleotide (170), penta-nucleotide (63) and hexa-nucleotide (50). An additional 718 SSRs were classified as SSR motifs in compound form; that is, the SSRs contained two or more repeat types separated by none to any number of base pairs. SSR motifs with five repeats (30%) were the most common, followed by six (29%), seven (15%), eight (8%), nine (4%) and ten (3%). The most frequent SSR motif was TC/GA (2440; 18%), followed by AG/CT (2384; 18%), TCT/AGA (841; 6%), GAA/TTC (776; 6%), CTT/AAG (711; 5%) and GAT/ATC (468; 3%), while GC/GC (2) was the least frequent SSR motif (Table 3). Most of these values are in agreement with a previous report of a radish RNA-seq dataset [13] and reports of EST analysis from other plant species, including watermelon [33]. Of the 12,403 SSR-containing unigenes, three combinations of primer pairs were designed for each of the 11,282 SSR motifs that had sufficient flanking sequences.

**Table 3:** Statistics of radish simple sequence repeats (SSRs)

**Nucleotide type**	**No. SSR**	**Repeat type**	**No. SSR**	**Motif type**	**No. SSR**
Di-nucleotide	5,918	5	4,117	TC/GA	2,440
Tri-nucleotide	6,651	6	3,959	AG/CT	2,384
Tetra-nucleotide	170	7	1,992	TCT/AGA	841
Penta-nucleotide	63	8	1,067	GAA/TTC	776
Hexa-nucleotide	50	9	602	CTT/AAG	711
Compound form	718	10	340	GAT/ATC	468

Since the ESTs described in this study were derived from 18 different accessions, including 14 cultivated (*R. sativus*) and 4 wild (*R. raphanistrum*) accessions, it is expected that SNPs would be highly abundant in this radish EST dataset. Using very stringent criteria (see methods), a total of 28,758 high-quality SNPs were detected in 4,764 unigenes. Of these, 15,029 were transitions, 10,051 were transversions, and 3,678 were single-base indels (Table 4). The identified SSRs and SNPs and their associated information are available at RadishBase [34] and provide a resource of valuable molecular markers to facilitate radish breeding and research.

**Table 4:** Statistics of radish single nucleotide polymorphisms (SNPs)

**SNP**	**No. SNPs**	**Type**	**Total**
A ↔ G or C ↔ T	15,029	Transition	15,029
A ↔ C	2433	Transversion	10,051
A ↔ T	2828		
C ↔ G	2276		
G ↔ T	2514		
T ↔ -	1288	Indel	3.678
A ↔ -	1172		
C ↔ -	611		
G ↔ -	607		

### Phylogenetic relationship analysis

**Figure 4:**
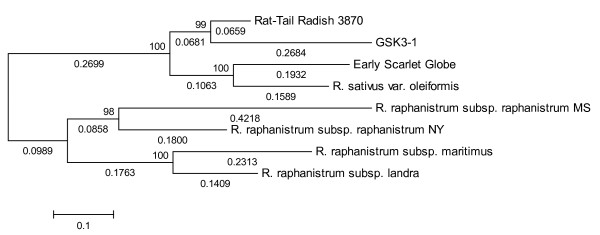
**Neighbor-joining tree of radishes.** Numbers above the branches denote the bootstrap values.

Phylogenetic analysis using DNA markers is not only an important tool to study the evolutionary relationships between organisms, at many levels, but also a technique that gives much deeper insight into the mechanism of maintenance of polymorphic alleles in populations [35]. To analyze the phylogenetic relationships of different *Raphanus* accessions, a neighbor-joining phylogenetic tree was constructed for eight accessions that had sufficient ESTs for the analysis, using a subset of 1,800 SNPs that had information derived from all eight accessions (Figure 4). The eight accessions were clearly separated into two groups: the first group included four accessions belonging to cultivated radish (*R. sativus*) and the second group included four wild radish accessions (*R. raphanistrum*). In the *R. sativus* group, Rat-Tail 3870 (var. *caudatus*), which is not an edible root variety but rather is used for its slender and edible seedpods, showed a closer phylogenetic relationship with GSK 3-1 (var. *hortensis*), which is a selfed progeny from a leading Japanese variety of R. *sativus* known as Utsugi-Gensuke [32], which has a long white root [36]. A close phylogenetic relationship was observed between Early Scarlet Globe (var. *radiculus*), known for its globular shape and white fleshy roots, and var. *oleiformis*, a fodder or oilseed radish (Figure 4). In the wild radish group, two accessions of subsp. *Raphanistrum* formed a sub-group, while subsp. *maritimus* and subsp. *landra* clustered together. Currently phylogenetic relationships between different radish genotypes remain largely uncertain. Lewis-Jonas et al. [18] proposed that a variant of the raphanistrum-landra complex might be the wild ancestor of the cultivated radish, while other studies suggested that the cultivated radish displayed multiple origins [37-39]. In the present study, a phylogenetic analysis based on 1,800 SNP markers strongly supported the proposition that the four radish cultivars share the same ancestor, which might originate from one subspecies of *R. raphanistrum* or the complex of the three subspecies. However, further studies are required to definitively establish the phylogenetic relationship between cultivated and wild radishes.

## Conclusion

The analysis of approximately 315,000 radish ESTs collected from the NCBI dbEST database was presented here. These ESTs were assembled *de novo* into 85,083 unigenes and functionally annotated. Comparative analysis between radish ESTs and other plant genome sequences revealed a number of highly conserved gene families across dicotyledonous and monocotyledons plants, as well as gene families that are specific to members of the Brassicaceae and to radish. Two recent WGD events were identified in radish; one before and one after the divergence of radish and *Brassica rapa*. In addition, the identified 13,570 SSRs and 28,758 high-quality SNPs represent valuable molecular markers and can be widely used in linkage map construction and the genetic mapping of QTLs associated with important agronomic traits. Based on 1,800 identified SNPs, the phylogenetic relationships between different *Raphanus* species were analyzed to investigate the evolutionary history of radish. The comprehensive analysis of *Raphanus* ESTs presented in this study will not only facilitate the annotation of the radish genome, which is currently being sequenced, but also provide a valuable resource for marker assisted breeding programs and further functional and comparative genomics analyses.

## Methods

### EST processing and assembly

*Raphanus* ESTs were collected from the NCBI dbEST database [22]. The EST sequences were first screened against the NCBI UniVec database [40], *E. coli* genome sequences and ribosomal RNA sequences using SeqClean [41], to remove possible contamination of these sequences. The ESTs were further processed to remove low quality (containing >3% Ns) and adaptor sequences. The resulting high-quality cleaned ESTs were assembled into unigenes using the iAssembler program, with default parameters [42].

### Functional annotation

The radish unigenes were functionally annotated by comparing their sequences against GenBank non-redundant (nr), UniProt (TrEMBL and SwissProt) and Arabidopsis protein databases using the BLAST program, with an E-value cutoff of 1e-5. Gene ontology (GO) terms were assigned to each unigene based on the GO terms annotated to its corresponding homologs in the UniProt databases. GO annotations of radish unigenes were then mapped to a list of plant-specific GO slim ontologies [43] and these GO slim terms were used to functionally classify radish unigenes.

### Comparative genomics analysis

The radish unigenes were compared to protein databases of four plant species, *Brassica rapa*, *A. thaliana*, *Vitis vinifera* (grape) and *Oryza sativa* (rice) using the BLAST program with an E-value cutoff 1e-5. Orthologous groups of protein sequences were identified using the orthoMCL program [44]. Venn diagram showing the distribution of shared and specific gene families among the five species was generated using the online Venn Diagrams program [45]. GO terms associated with the radish unigenes that were significantly enriched in each list of specific orthologous groups were identified using GO: TermFinder perl module [46] implemented in RadishBase [34], with a cutoff of corrected p values (False Discovery Rate, FDR) of no more than 0.01.

### Whole-genome duplication (WGD) analysis in radish

To investigate potential WGD events in radish, gene families were constructed and the rate of synonymous substitutions (Ks) between each homologous gene pair was calculated using the methods described in Jiao et al. [5] and Shi et al. [4]. Specifically, radish unigenes were first translated into proteins using ESTScan [47] with a matrix built from the coding sequences (CDS) of *Brassica rapa*[48]. Only the translated protein with best matches to the *B. rapa* proteins was kept if a particular radish unigene had multiple translated protein sequences. Translated proteins with < 100 amino acids were not included in the analysis. Radish CDS sequences were then aligned to themselves using BLASTN with an E-value cutoff of 1e-5 and based on the alignments redundancies of radish translated protein sequences caused by alternative splicing were removed and orthologues from different sub-species according to the following rules: 1) If the two CDS had an alignment longer than 600 bp and > 95% amino acid sequence identity, the shorter sequence was removed; 2) If the two CDS were both shorter than 600 bp and the alignment was longer than 300 bp, with >95% amino acid sequence identity, the shorter sequence was removed; 3) If the aligned region covered 95% of one of the gene pair with no less than 95% amino acid sequence identity, the shorter CDS was removed.

Orthologous groups were then constructed using OrthoMCL [44] based on the all-to-all BLASTP results of protein sequences from radish, *A. thaliana*[49], *Brassica rapa*[48], and papaya [50], with an E-value cutoff of 1e-20. The proteins in each orthologous group were aligned using MUSCLE with default parameters [51], and the alignments were then trimmed using trimAL [52] to remove poorly aligned regions, using the parameter “-automated1”. The resulting trimmed protein alignments were then converted to the corresponding CDS alignments, which were used for Ks calculations. For the Ks calculation between species, the orthologous groups with one gene from each of the four plant species were used. For the Ks calculation within species, homologous pairs with the best reciprocal matches were used. The Ks values were calculated using the yn00 program in the PAML package under the F3x4 model [53]. Only Ks values >0 and <3 were used in the analysis.

The speciation events as well as WGD events were dated based on the Ks values. The average substitution rate (r) used to calibrate the age of the considered genes was calculated based on the fossil record time interval (54-90 million years ago) of Capparales, as previously reported [54,55]. The time (T) was then estimated using the formula T = Ks/r [56,57].

### SSR and SNP identification

The complete set of radish unigene sequences were screened for the presence of SSR motifs using the MISA program [58]. The minimum repeat was six for dinucleotide and five for tri-, tetra-, penta and hexa-nucleotide. Primer pairs were designed for SSR motifs that had sufficient flanking sequences using the Primer3 program [59].

SNPs in the EST sequences among the different radish cultivars were identified as described in Clepet et al. [60]. Briefly, polymorphisms were first identified with PolyBayes [61] and only those with at least 2× coverage for each accession and no same bases at the potential SNP site between the two compared accessions were considered. Detailed information related to all radish SSRs and SNPs is publicly available at the RadishBase [34].

### Phylogenetic analysis of radish germplasm

Among the 18 radish accessions used for EST generation, eight were selected to determine their phylogenetic relationship as they had a sufficient number of ESTs for the analysis. These eight accessions were Rat-Tail Radish 3870, GSK3-1, Early Scarlet Globe, *R. sativus* var. *oleiformis*, *R. raphanistrum* subsp. *raphanistrum* MS and NY, *R. raphanistrum* subsp. *maritimus* and *R. raphanistrum* subsp. *landra*. A neighbor-joining phylogenetic tree was constructed with MEGA5 [62], using a total of approximately 1,800 SNP markers that had information in all eight accessions. Trees were bootstrapped 1,000 times to assure confidence in topology.

## Competing interests

The authors declare that they have no competing financial interests.

## Authors’ contributions

ZF and XL conceived and designed the study. DS, HS, MH, YZ, YQ and ZF performed the data analysis. DS, HS and ZF wrote the manuscript. All authors approved the final manuscript.

## Supplementary Material

Additional file 1 **Summary of radish transcription factors.** The table provides the number of radish transcription factors identified in each family. Click here for file

Additional file 2 **Radish transcription factors.** The table provides the list of all identified putative radish transcription factors. Click here for file

Additional file 3 **GO terms enriched in Brassicaceae-specific gene families.** The table provides the list of enriched GO terms identified from radish unigenes that are in gene families specific to the Brassicaceae family. Click here for file

Additional file 4 **GO terms enriched in radish-specific gene families.** The table provides the list of enriched GO terms identified from radish-specific unigenes. Click here for file

Additional file 5 **Distribution of synonymous nucleotide substitution (Ks) rates.** This figure shows the distribution of synonymous nucleotide substitution (Ks) rates between homologous gene pairs within Arabidopsis (yellow), papaya (black) and between *Brassica rapa* and Arabidopsis (cyan) and *Brassica rapa* and papaya (red). Click here for file
