# Supplementary material for: Comprehensive analysis of expressed sequence tags from cultivated and wild radish (Raphanus spp.)
Source: BMC Genomics. 2013 Oct 21;14:721. doi: 10.1186/1471-2164-14-721 (PMC3816612; doi:10.1186/1471-2164-14-721)
Supplement: Additional file 5 — Distribution of synonymous nucleotide substitution (Ks) rates. This figure shows the distribution of synonymous nucleotide substitution (Ks) rates between homologous gene pairs within Arabidopsis (yellow), papaya (black) and between Brassica rapa and Arabidopsis (cyan) and Brassica rapa and papaya (red). [file 1471-2164-14-721-S5.pdf]

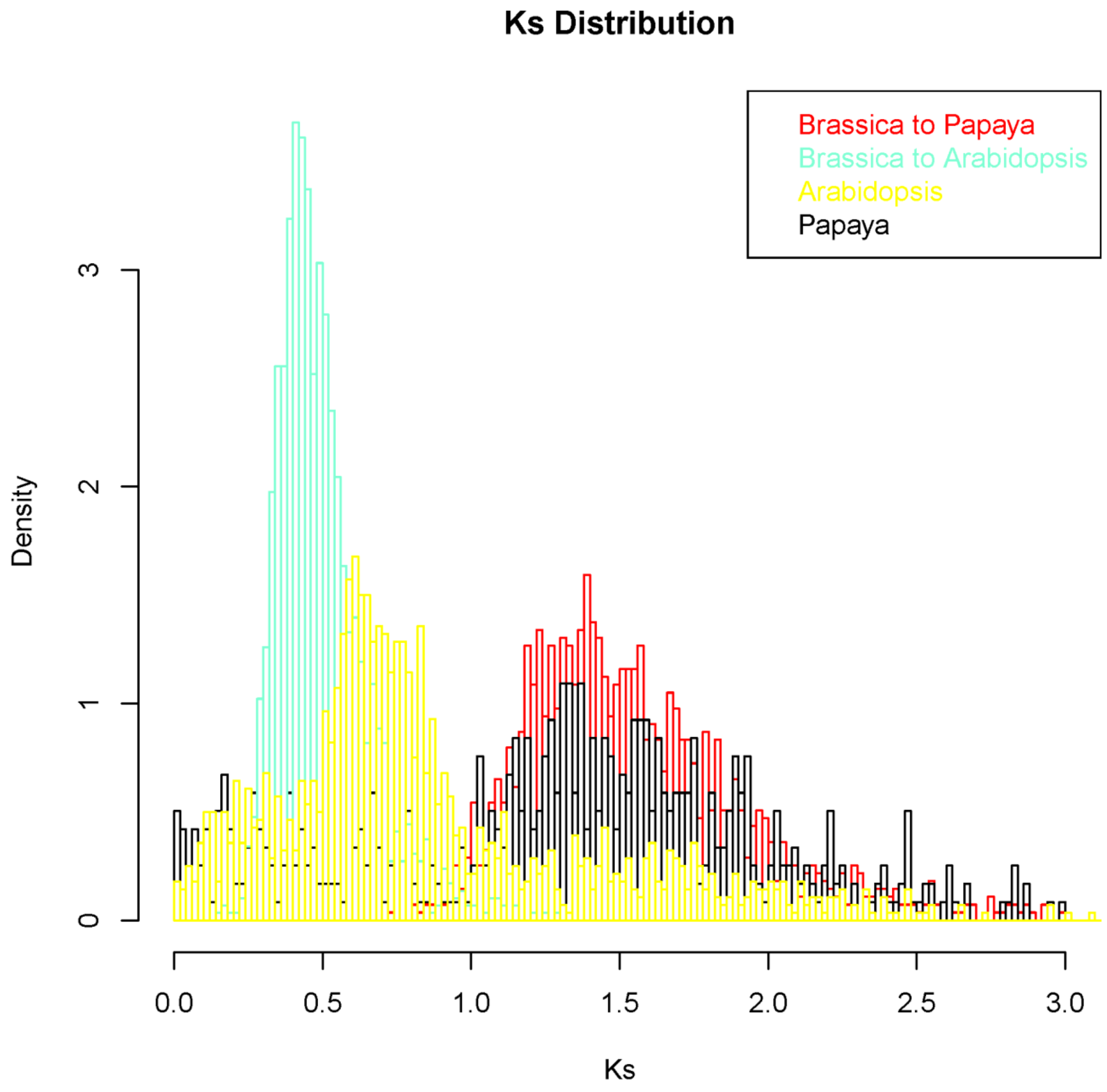

Additional file 5. Distribution of synonymous nucleotide substitution (Ks) rates between homologous gene pairs within Arabidopsis (yellow), papaya (black) and between *Brassica rapa* and Arabidopsis (cyan) and *B. rapa* and papaya (red).
